# A sewing needle in liver: a case report and review of the literature

**DOI:** 10.1186/1757-1626-2-6520

**Published:** 2009-06-01

**Authors:** Quan Zhou Feng, Jie Wang, Hong Sun

**Affiliations:** 1 Clinical Division of Nanlou, Chinese PLA General HospitalFuxing Road 28, Beijing, 100853China; 2 Department of Ultrasound, Chinese PLA General HospitalFuxing Road 28, Beijing, 100853China; 3 Department of Radiology, Chinese PLA General HospitalFuxing Road 28, Beijing, 100853China

## Abstract

**Introduction:**

Hepatic foreign bodies are quite rare. A sewing needle as a hepatic foreign body in an old woman is very rare and the managements have been varied.

**Case presentation:**

An old woman was incidentally found to have a sewing needle in her liver on abdominal X-ray. The sewing needle was kept stable in her liver after two years of follow-up. Eleven cases of sewing needle in the liver were reviewed.

**Conclusion:**

Sewing needle as a foreign body in the liver is rare. In general, the sewing needle should be removed through laparotomy or laparoscopy, but a stable and uncomplicated sewing needle in the liver need not be removed.

## Introduction

Hepatic foreign bodies are quite rare. A sewing needle (SN) as a hepatic foreign body in an old woman is very rare. The managements have been varied. Herein, we report a 76-year-old woman with a SN in her liver, which was incidentally found on abdominal X-ray during hospitalization for treatment of anemia.

## Case presentation

**Figure 1.**
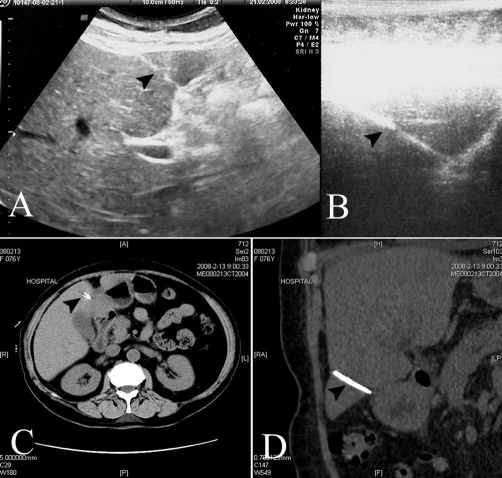
The sewing needle in ultrasound examination and computerized tomographic scan. **A**: Ultrasound showed a 3.5 cm long needle-like metal object in left lobar of the liver, the arrow points to the needle; **B**: Partial magnification of the needle in panel A; **C**: Computerized tomographic scan validated the metal object as a SN, the arrow points to the pore in the needle; **D**: Reconstructed computerized tomogram of the needle in liver.

A 76-year-old woman was hospitalized due to the complaint of fatigue. On evaluation, she was found to have anemia. Full blood count revealed the following findings: red blood cell count, 2.33 × 10^12^/L; hemoglobin, 76 g/L; mean corpuscular volume, 101.7 (normal: 80-100) fl; mean corpuscular hemoglobin, 32.6 (normal: 27-34) pg; reticulocytes, 6.4%; white blood cell count, 6 × 10^9^/L; and platelets, 371 × 10^9^/L. The patient was found to have a serum iron content of 6.4 (normal: 7-32) μmol/L and unsaturated iron binding capacity of 56.1 (normal: 31-51) μmol/L. Liver and kidney function tests were normal. Abdominal x-ray incidentally revealed a metal needle in her superior abdominal area. She had no history of inadvertently swallowing a metal needle and no history of abdominal operation but she recalled that she was acupunctured by a witch doctor because of epigastric pain more than twenty years ago. She was not aware that a needle was left in her body. She had not had epigastric pain for many years. Ultrasound examination showed a 3.5 cm long, needle-like, metal object in the left lobar of the liver (Figure 1: Panels A and B). Computerized tomographic scan validated the metal object as a SN (Figure 1: Panels C and D). She was diagnosed with nutritional anemia and a SN in the liver. After supplement of iron, vitamin B12, and folacin, her haemoglobin was recovered to a near normal level (92 g/L). She was discharged without removal of the needle because the needle had been in her body for many years, without harm to her health. The needle was kept stable in her liver after two years of follow-up.

## Discussion

SN as a foreign body in the liver is rare, so far only 11 cases have been reported in English literature [1-11] (Table 1). The patients have been psychiatric cases [6,9], a pediatric population [8], or ordinary adults [7,10,11] that accidentally swallowed a foreign object. Among them, five cases were children under 14 years old [1,2,4,5,8]. In the six cases of adult patients, five cases were women, which might be attributed to a SN being used more often by a woman than a man. The way by which the SN enters into liver may be transcutaneous, but in 9 of 11 cases reviewed, the SNs migrated to liver through the gastrointestinal tract after the SNs were, inadvertently [6-8,10] or intentionally [9], ingested. The two patients with a SN transcutaneously entering the liver had no clear history. The entering pathway was confirmed by operation in one case [5], and the other had the habit of sticking needles into her body [3], which suggested the needle penetrated into liver transcutaneously.

**Table 1:** Summarised data on eleven cases of hepatic sewing needles

Reporter	Age	Sex	Diagnosis of needle	Route to liver	Location	Hepatic Abscess	Treatment method
Abel RM, et al. [1]	11 months	Male	Incidentally	Stomach	Left lobe	Yes	Laparotomy
Crankson SJ [2]	2 years	Male	Incidentally	?	Right lobe	No	No treatment
Saviano M, et al. [3]	65 years	Female	Incidentally	Transcutaneous	Left lobe	No	Laparoscopy
Le Mandat-Schultz A, et al. [4]	11 months	Male	Swallowing history	Gastrointestinal tract?	Right lobe	No	Laparoscopy
Nishimoto Y, et al. [5]	1 year	Male	Incidentally	Transcutaneous	Left lobe	No	Laparotomy
Roca B[6]	85 years	Female	Swallowing history, senile dementia	Gastrointestinal tract?	Left lobe	No	No treatment
Chintamani, et al. [7]	26 years	Male	Incidentally, fever	Duodenum	Right lobe	Yes	Laparotomy
Azili MN, et al. [8]	14 years	Female	Swallowing history, epigastric pain	Stomach	Right lobe	No	Laparotomy
Lanitis S, et al. [9]	35 years	Female	Swallowing history	Duodenum	Left lobe	No	Laparoscopy
Rahalkar MD, et al. [10]	23 years	Female	Swallowing history	Gastrointestinal tract	Left lobe	No	No treatment
Ward A, et al. [11]	20 years	Female	Swallowing history	Duodenum	Left lobe	No	Laparotomy

The patients with a SN in the liver usually have no obvious symptoms except mild epigastric pain which is often neglected. Only two of the eleven cases reviewed were complicated with hepatic abscess, which was secondary to the SNs, migrated from the alimentary tract. The clinical picture in these cases included fever with chills and rigors, abdominal pain, vomiting, and jaundice, and patients needed to be treated with surgical drainage [1,7]. In most cases, the SN in the liver was incidentally detected by X-ray during medical examination.

The management of a SN depends on its location, progression, and existence of any complication. Retrieval methods of a SN include laparotomy [1,5,7,18,11] and laparoscopy [3,4,9]. In most cases (8/11), the SN was surgically removed to avoid complication. Asymptomatic patients without complication need not be treated with immediate operation, but the patient should be followed up. If the SN is stable in the liver without movement, the needle need not be retrieved [6,10].

The current patient did not know when the sewing entered into her liver, which was incidentally found and presented no symptoms. The SN remained stable in the liver after two years of follow-up, so was not removed.

## Conclusion

A SN as a foreign body in the liver is rare. In general, the SN should be removed through laparotomy or laparoscopy, but stable and uncomplicated SN in the liver need not be removed.

## Abbreviations

SN: sewing needle

## References

[bib-001] Abel RM, Fischer JE, Hendren WH (1971). Penetration of the alimentary tract by a foreign body with migration to the liver. Arch Surg.

[bib-002] Crankson SJ (1997). Hepatic foreign body in a child. Pediatr Surg Int.

[bib-003] Saviano M, Melita V, Tazzioli G, Farinetti A, Drei B (2000). Videolaparoscopic removal of a foreign body from the liver. Eur J Surg.

[bib-004] Le Mandat-Schultz A, Bonnard A, Belarbi N, Aigrain Y, De Lagausie P (2003). Intrahepatic foreign body laparoscopic extraction. Surg Endosc.

[bib-005] Nishimoto Y, Suita S, Taguchi T, Noguchi S, Ieiri S (2003). Hepatic foreign body - a sewing needle - in a child. Asian J Surg.

[bib-006] Roca B (2003). A sewing needle in the liver. South Med J.

[bib-007] Chintamani, Singhal V, Lubhana P, Durkhere R, Bhandari S (2003). Liver abscess secondary to a broken needle migration--a case report. BMC Surg.

[bib-008] Azili MN, Karaman A, Karaman I, Erdogan D, Cavusoglu YH, Aslan MK, Cakmak O (2007). A sewing needle migrating into the liver in a child: case report and review of the literature. Pediatr Surg Int.

[bib-009] Lanitis S, Filippakis G, Christophides T, Papaconstandinou T, Karaliotas C (2007). Combined laparoscopic and endoscopic approach for the management of two ingested sewing needles: one migrated into the liver and one stuck in the duodenum. J Laparoendosc Adv Surg Tech A.

[bib-010] Rahalkar MD, Pai B, Kukade G, Al Busaidi SS (2003). Sewing needles as foreign bodies in the liver and pancreas. Clin Radiol.

[bib-011] Ward A, Ribchester J (1978). Migration into the liver by ingested foreign body. Br J Clin Pract.

